# Interesting Case of Anterior Communicating Artery Aneurysm Turns Out to Be a Ruptured Fenestrated A1 Segment Aneurysm With Concomitant Multiple Aneurysms: A Case Report and Literature Review

**DOI:** 10.7759/cureus.68341

**Published:** 2024-08-31

**Authors:** Nitin V Naikwade, Nitin N Dange, Naren Nayak, Ashvini P Mahamuni, Prakash Palave

**Affiliations:** 1 Neurosurgery, Lokmanya Tilak Municipal Medical College Sion, Mumbai, IND; 2 Neurosurgery, Lokmanya Tilak Municipal Medical College and General Hospital, Mumbai, IND

**Keywords:** types of fenestrated a-1 segment aneurysm, aneurysm clipping, multiple aneurysm, fenestrated a-1 segment, arterial fenestration

## Abstract

The luminal separation of the vessel into two distinct, parallel channels that reunite distantly is known as fenestration. There is a correlation between the development of an aneurysm and the proximal portion of fenestration, although fenestrations are typically regarded as a variant of normal anatomy. We report an interesting case of an anterior communicating artery (a-comm) aneurysm, which turns out to be a ruptured fenestrated A1 segment aneurysm after digital subtraction angiography with concomitant multiple aneurysms treated by clipping along with case report and literature review.

## Introduction

The luminal separation of the vessel into two distinct, parallel channels that reunite distantly is known as fenestration. There is a correlation between the development of an aneurysm and the proximal portion of fenestration, although fenestrations are typically regarded as a variant of normal anatomy [1]. Depending on the degree of embryological fusion, each channel might possess a shared adventitial layer, distinct endothelial and muscular layers, and varying sizes. It is proposed that focal defects in the media layer close to the locations of channel divergence and convergence are secondary to this. In the horizontal portion of the anterior cerebral artery (A1), cerebral aneurysms are quite rare (0.88%) [2]. The development of A1 fenestrations is less understood than that of basilar and vertebral artery fenestrations, even though there are currently few reports on fenestrations in the A1 segment. The majority of fenestrated A1 aneurysms have been surgically removed.

We report an interesting case of an anterior communicating artery (a-comm) aneurysm, which turns out to be a ruptured fenestrated A1 segment aneurysm after digital subtraction angiography (DSA) with concomitant multiple aneurysm treated by clipping.

## Case presentation

**Figure 1:**
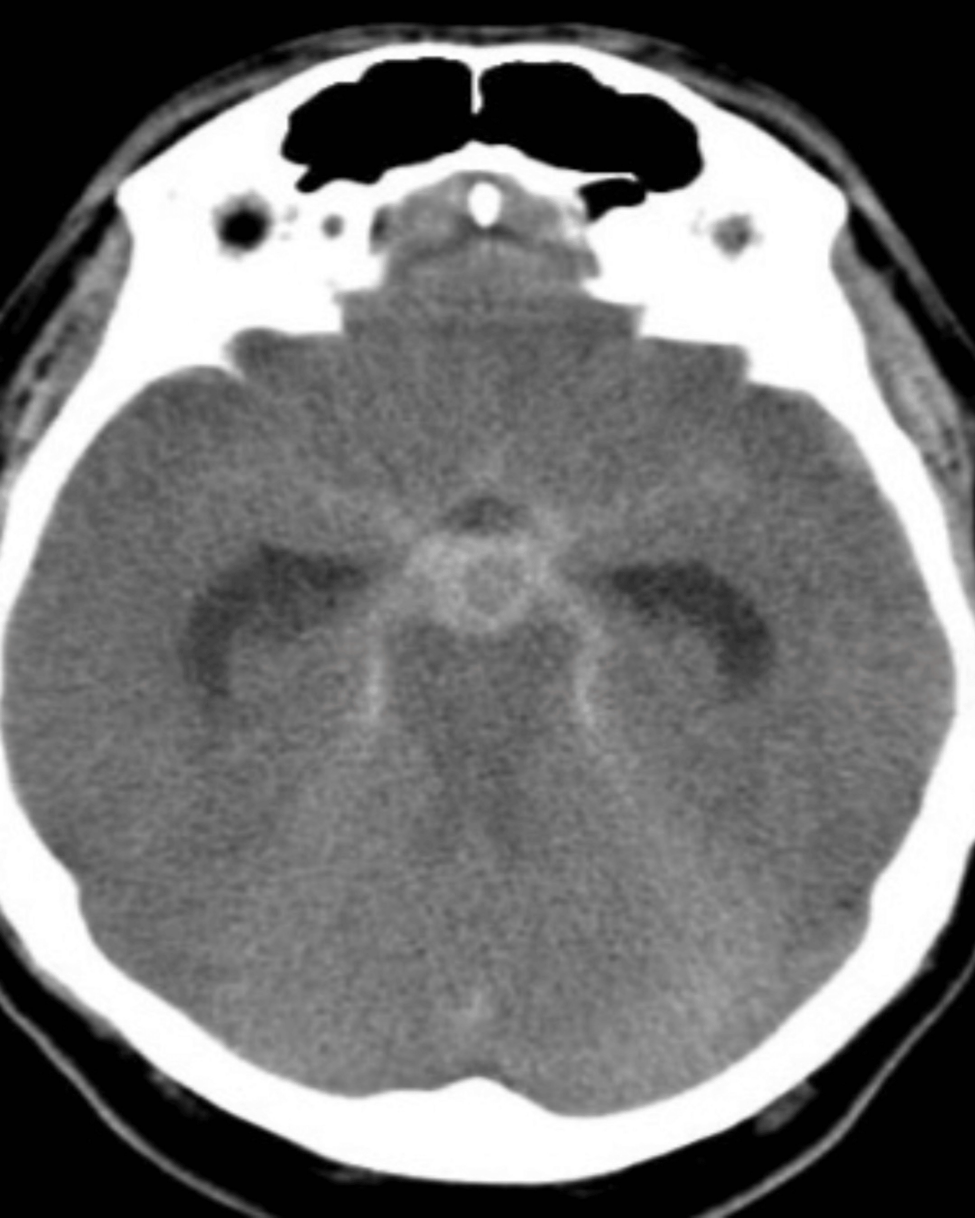
Non-contrast CT suggestive of subarachnoid hemorrhage in Sylvian and basal cisterns and ruptured anterior communicating artery aneurysm.

A 58-year-old female patient was referred to us with the chief complaint of giddiness and loss of consciousness while working, along with vomiting and a holocranial headache. There was no history of hypertension or other comorbidities. Came with non-contrast CT (NCCT) brain and CT brain angiography, which was done outside with reports suggestive of subarachnoid hemorrhage in Sylvian and basal cisterns and ruptured a-comm aneurysm (Figure 1).

**Figure 2:**
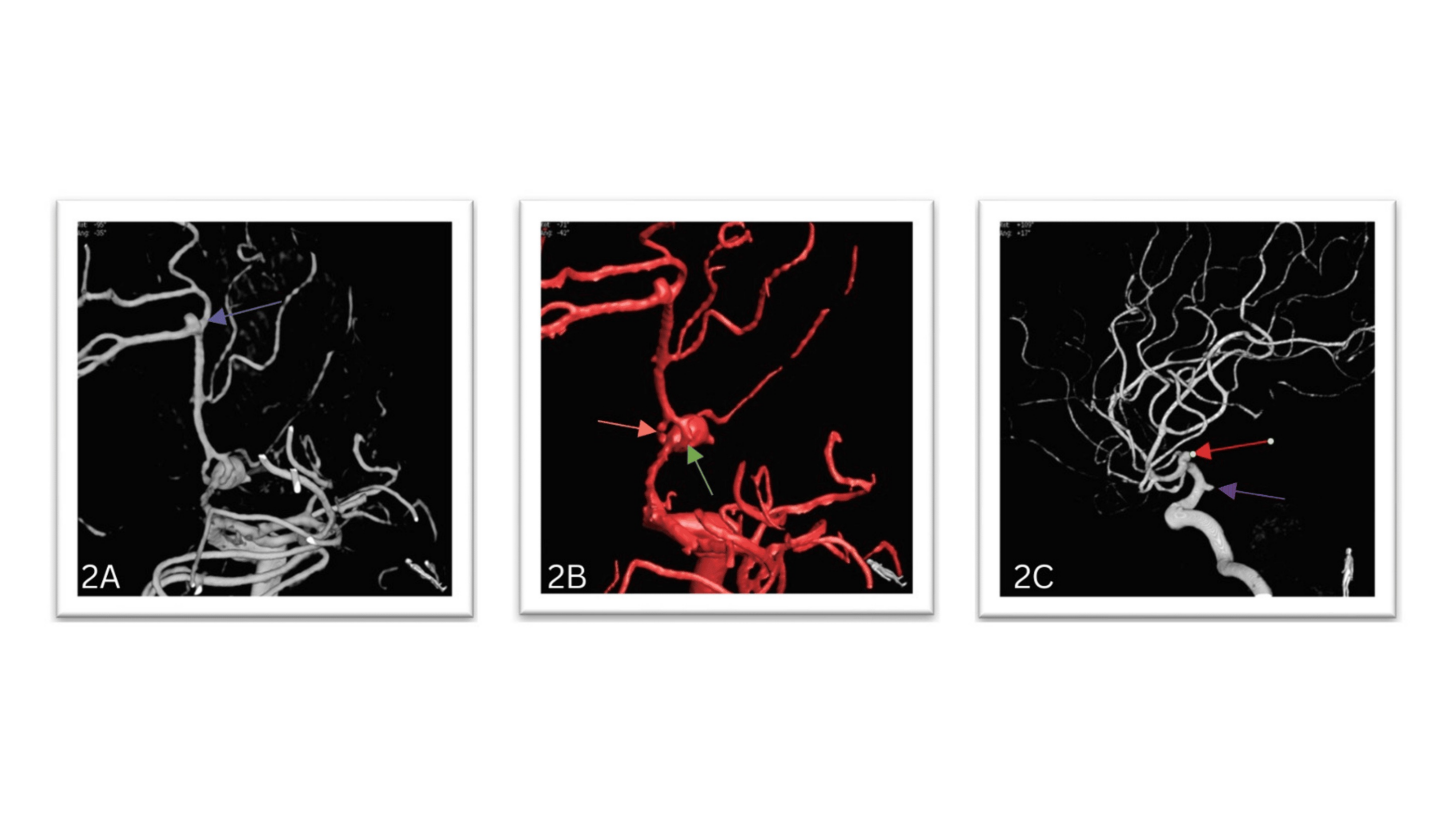
(A) Saccular aneurysm at the proximal end of the fenestrated right A1 segment, saccular aneurysm at the right distal anterior cerebral artery (blue Arrow). (B) The medial trunk of duplicated A1 (orange arrow), the lateral trunk of duplicate A1 (green arrow). (C) Saccular aneurysm at the left M1 segment of the middle cerebral artery (red arrow), the left posterior communicating artery (p-comm) segment aneurysm (purple arrow).

A three-dimensional DSA performed at our institution revealed a neck of 2.8 mm saccular aneurysm at the proximal end of the fenestrated right A1 segment, a neck of 2 mm saccular aneurysm at the right distal anterior cerebral artery (DACA), and a neck of 2.6 mm saccular aneurysm at the left M1 segment of the middle cerebral artery and the left posterior communicating artery (p-comm) segment (Figures 2A-2C).

**Figure 3:**
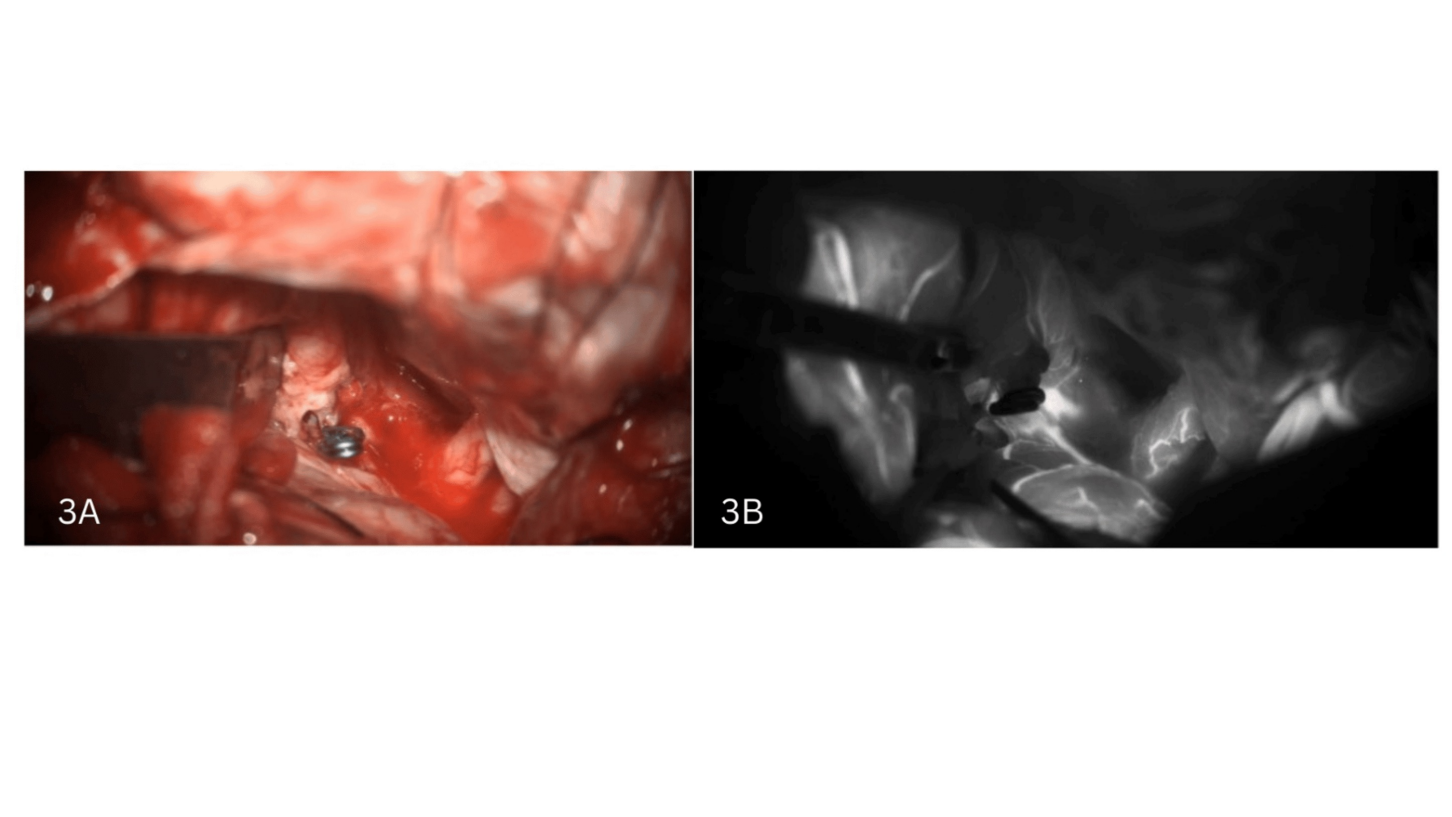
(A) The aneurysm was clipped successfully using a single straight clip of size 7 mm. (B) Post-clipping intraoperative indocyanine green (ICG) videography showed complete obliteration of the aneurysm and the well-preserved patency of both the lumens of fenestration.

The patient was informed about endovascular and surgical management and its risks and benefits. The patient chose a surgical procedure and underwent surgical clipping of the right A1 fenestrated using a fronto-temporo-parietal (FTP) craniotomy and right DACA aneurysm using an inter-hemispheric approach. On surgical exploration, the aneurysm identified was just proximal to the right fenestrated A1 segment. The aneurysm was clipped successfully using a single straight clip of size 7 mm (Figure 3A). Post-clipping intraoperative indocyanine green (ICG) videography showed complete obliteration of the aneurysm and the well-preserved patency of both the lumens of fenestration (Figure 3B).

**Figure 4:**
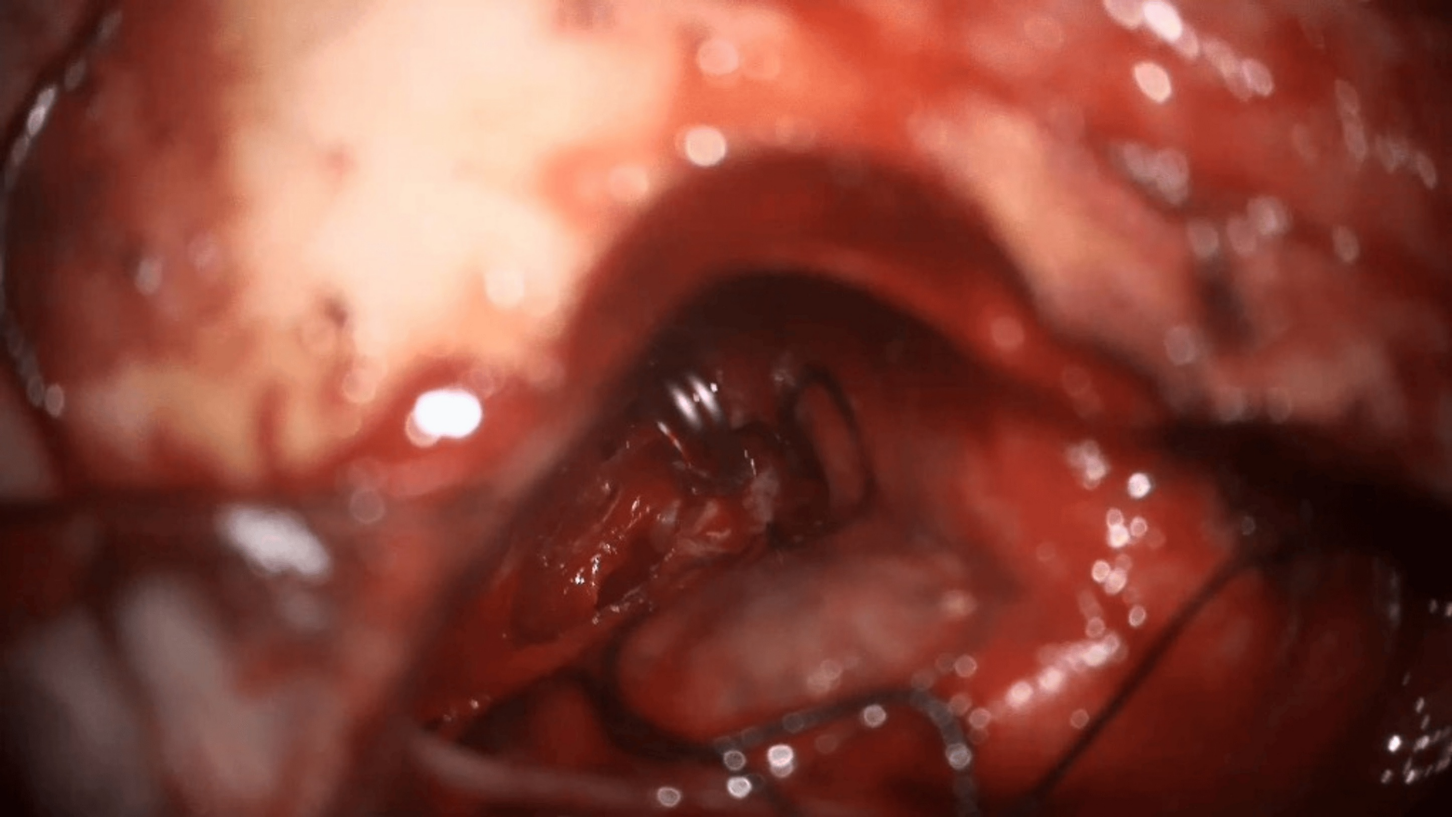
Right distal anterior cerebral artery aneurysm clipped via intra-hemispheric approach using one clip, size 7 mm straight.

Right DACA aneurysm clipped via intra-hemispheric approach using one clip, size 7 mm straight (Figure 4).

The postoperative period was uneventful, and the patient was discharged with a good recovery.

## Discussion

Fenestration is the partition of the arterial lumen into separate channels, each of which has its own endothelium and muscular layer. Nevertheless, these distinct arterial channels may or may not share the adventitia [3]. The vertebrobasilar arteries have a higher prevalence of fenestrations, followed by the middle cerebral artery and the anterior cerebral artery [4]. In most anterior cerebral artery anatomical investigations, the incidence of fenestration of the A1 segment ranges from 0% to 4% [5]. A review of the literature reveals that 22 cases of this type have been previously reported (Table 1). There were 12 male and 10 female patients, with a mean age of 48 years. Eighteen (78.22%) cases of ruptured aneurysms and five (21.7%) cases of unruptured aneurysms were present. Analysis revealed a right-to-left ratio of 15:8, with 65% of patients reporting favorable outcomes and 13% resulting in mortality. Multiple aneurysms were reported in three (13%) cases.

**Table 1:** Literature review ACA, anterior cerebral artery; MCA, middle cerebral artery; PCA, posterior cerebral artery

Serial no.	Age	Sex	Side	Site	Size (mm)	Projection	Type	Multiplicity	Related vascular anomaly	Management	Outcome	Reference
1	43	M	Right	Proximal end	NA	Superomedial	Ruptured	-	-	Aneurysm neck clipping	Death	Yamada et al [7].
2	41	M	Right	Proximal end	NA	Superomedial	Ruptured	-	-	Aneurysm neck clipping	Death	Korosue et al. [8]
3	50	F	Left	Proximal end	NA	Superomedial	Ruptured	-	-	Aneurysm neck clipping	NA	Handa et al. [9]
4	56	M	Right	Proximal end	NA	Superomedial	Ruptured	-	VA fenestration	Aneurysm neck clipping	NA	Minakawa et al. [10]
5	41	M	Right	Proximal end	5.0	NA	Ruptured	-	-	-	Death	Wakabayashi et al [11]
6	38	M	Right	Proximal end	6.5	NA	Ruptured	-	-	-	Partial recovery	Wakabayashi et al [11]
7	33	M	Right	Proximal end	NA	Superomedial	Ruptured	-	Azygos ACA, contralateral A1 fenestration	Aneurysm neck clipping	Good recovery	Friedlander et al [12]
8	50	F	Right	Proximal end	NA	Superomedial	Ruptured	-	Contralateral A1 aplasia	Aneurysm neck clipping	-	Kachhara et al. [13]
9	65	F	Left	Proximal end	NA	Inferior	Ruptured	-	-	Aneurysm neck clipping	-	Ogasawara et al [14]
10	68	M	Left	Medial limb	5.0	Inferior	Ruptured	-	Ipsilateral M1 aplasia	Aneurysm neck clipping	Good recovery	Taylor et al. [15]
11	52	F	Left	Proximal end	4.3	Superomedial	Ruptured	-	-	Aneurysm neck clipping	Good recovery	Wanibuchi et al [16]
12	78	F	Left	Lateral limb	NA	Superomedial	Unruptured	-	Azygos ACA, contralateral A1 hypoplasia	Aneurysm neck clipping	Good recovery	Ihara et al. [17]
13	49	M	Right	Proximal end	4.0	Superomedial	Ruptured	-	-	Aneurysm neck clipping	Good recovery	Leyon et al. [18]
14	50	F	Right	Proximal end	NA	Superomedial	Ruptured	-	Contralateral A1 fenestration	Aneurysm neck clipping	Good recovery	Aktüre et al. [19]
15	59	F	Right	Proximal end	2.9	Inferomedial	Ruptured	MCA aneurysm	Azygos ACA, contralateral A1 hypoplasia	Aneurysm neck clipping	Good recovery	Kwon et al. [20]
16	72	M	Left	Proximal end	10.0	Superomedial	Unruptured	-	-	Aneurysm neck clipping	Good recovery	Iwabuchi et al. [21]
17	73	M	Left	Proximal end	7.0	Superomedial	Unruptured	-	-	Aneurysm neck clipping	Good recovery	Iwabuchi et al. [21]
18	49	F	Right	Proximal end	8.0	Superomedial	Ruptured	-	Azygos ACA, contralateral A1 hypoplasia	Aneurysm neck clipping	Good recovery	Gill et al. [22]
19	62	M	Right	Proximal end	4.2	Medial	Unruptured	-	-	Aneurysm neck clipping	Good recovery	Mamadalive et al [23]
20	63	M	Right	Proximal end	NA	Medial	Unruptured	-	-	Aneurysm neck clipping	Good recovery	Mamadalive et al [23]
21	64	F	Left	Proximal end	2.9	Superomedial	Ruptured	-	-	Aneurysm neck clipping	Good recovery	Kubota et al. [24]
22	75	F	Right	Distal	NA	Superomedial	Ruptured	ICA unruptured aneurysm	Duplication right MCA, fetal right PCA	Conservative	Good recovery	Koh et al. [25]
23	58	F	Right	Proximal end	2.8	Superomedial	Ruptured	Left M1 MCA, left p-comm artery aneurysm	Right DACA aneurysm	Aneurysm neck clipping	Good recovery	Present case

According to a review of the literature, the patients with ruptured aneurysms had a mean age of 48 years. A total of 13% of patients with fenestrated A1 aneurysms had multiple aneurysms, which is approximately 15-20% of all patients with aneurysms having multiple aneurysms [6]. Thus, there may be no correlation between the development of multiple aneurysms and fenestrated A1 aneurysms. The case in this report had a 2.8 mm saccular aneurysm at fenestrated A1 of the right anterior cerebral artery, a 2 mm aneurysm at the right DACA, a 2.6 mm aneurysm at the left M1 segment of the middle cerebral artery, and the left p-comm segment.

**Figure 5:**
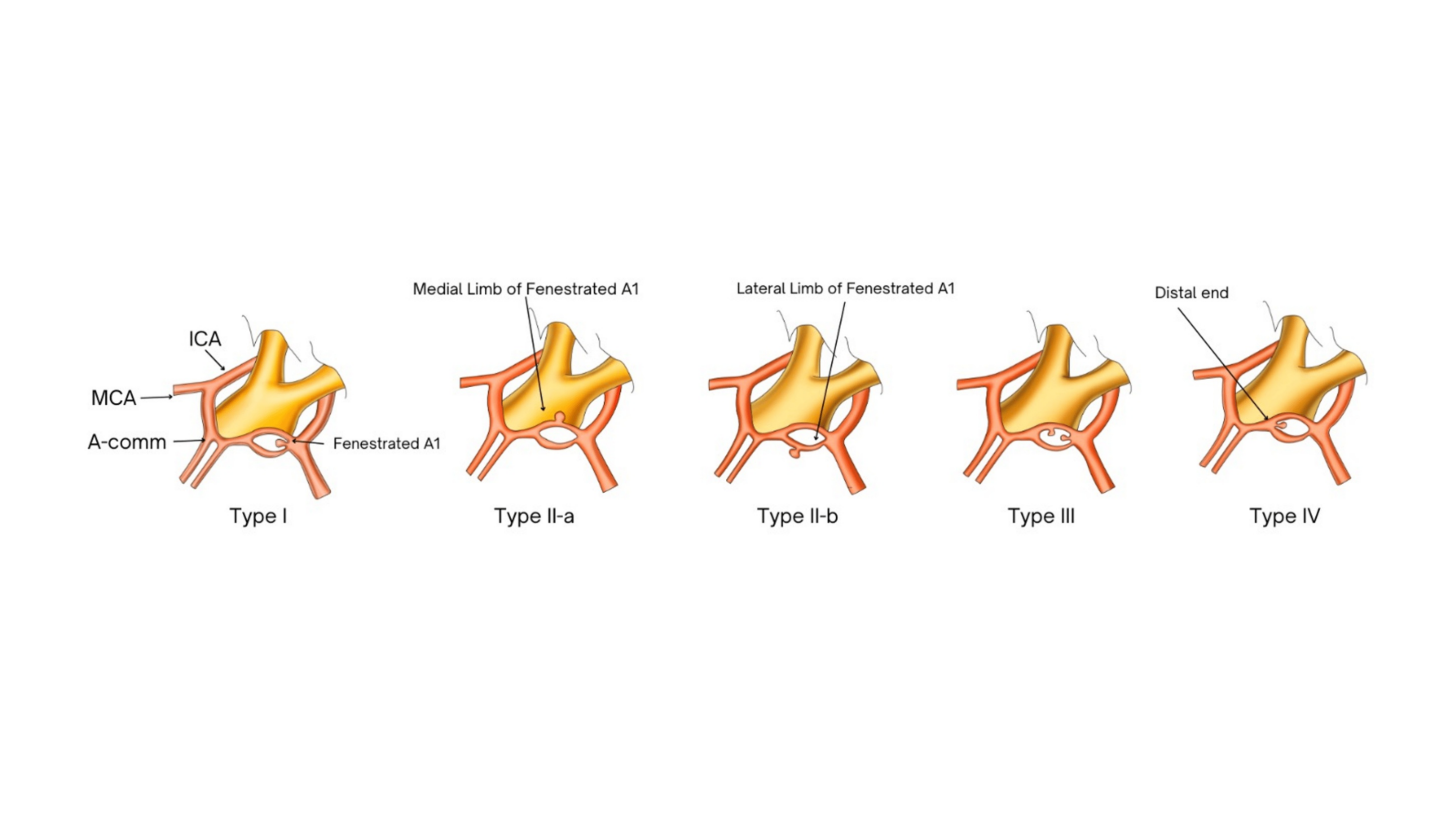
Classification of fenestrated A1 segment aneurysm. Type I: aneurysms on the proximal end of the fenestration. Type II: arising from the duplicated A1 limb; Type II-a: aneurysm arises from the medial limb of the duplicated A1; Type II-b: aneurysm arises from the lateral limb of the duplicate A1. Type III: multiple aneurysms within the fenestrated A1 limb. Type IV: aneurysms on the distal end of the fenestration. Animated 3D sketch made by Mr. Aditya Mahamuni with original idea from Dr. Nitin Naikwade

We propose an updated novel classification approach to further classify fenestrated A1 aneurysms based on anatomical features, building on the findings of Kwon et al. and Kubota et al. (Figure 5) [20,25]. Type I: aneurysms on the proximal end of the fenestration. Type II: arising from the duplicated A1 limb; Type II-a: aneurysm arises from the medial limb of the duplicated A1; Type II-b: aneurysm arises from the lateral limb of the duplicate A1. Type III: multiple aneurysms within the fenestrated A1 limb. Type IV: aneurysms on the distal end of the fenestration. A ruptured aneurysm arising from the distal end of A1 fenestration was reported by Koh et al. (2009) [25].

The fenestration makes the management of aneurysm more difficult because the A1 segment supplies the important structures in the brain, such as the optic chiasm, hypothalamus, caudate head, globus pallidus, anterior limb of the internal capsule, medial third of the anterior commissure, etc. [24]. Perlmutter and Rhoton et al. [6] described the majority of the normal A1 perforators as originating from the lateral surface of A1, so the origin of the A1 perforators in a fenestrated A1 segment with the location of aneurysm on the medial or lateral trunk is very important for planning and treatment. Fenestrated A1 segment aneurysms can be better understood with this new classification system and also help in tailored management in the form of open surgical or endovascular treatment.

## Conclusions

With detailed knowledge of the vascular anatomy of the fenestrated A1 segment aneurysm, the patient and aneurysm-specific management should be done. Aggressive treatment should be taken into consideration if an unruptured cerebral aneurysm is discovered because there have been studies suggesting that these aneurysms are prone to rupturing.
